# Bio-orthogonal crosslinking and hyaluronan facilitate transparent healing after treatment of deep corneal injuries with in situ-forming hydrogels

**DOI:** 10.1038/s41536-024-00385-9

**Published:** 2025-02-04

**Authors:** Fang Chen, Uiyoung Han, Thitima Wungcharoen, Youngyoon Amy Seo, Peter Le, Li Jiang, Nae-Won Kang, Euisun Song, Kyeongwoo Jang, David Mundy, Gabriella Maria Fernandes-Cunha, Sarah Heilshorn, David Myung

**Affiliations:** 1 Spencer Center for Vision Research, Byers Eye Institute, Stanford University School of Medicine, Palo Alto, CA USA; 2 VA Palo Alto Health Care System, Palo Alto, CA USA; 3 Materials Science and Engineering, Stanford University, Stanford, CA USA; 4 Chemical Engineering, Stanford University, Stanford, CA USA

**Keywords:** Preclinical research, Translational research

## Abstract

Corneal transplantation is the primary treatment for corneal blindness, affecting millions globally. However, challenges like donor scarcity and surgical complications remain. Recently, in situ-forming corneal stroma substitutes have emerged, offering potential solutions to these limitations. These substitutes enable liquid-to-hydrogel formation in situ, eliminating sutures and reducing complications. Here we performed a direct, side-by-side comparison of a composite hyaluronan-collagen (HA-Col) hydrogel crosslinked by either photochemistry or bio-orthogonal chemistry to ascertain the impact of reaction specificity on corneal wound healing. Testing in rodent and rabbit models suggests that composite HA-Col gels crosslinked by bio-orthogonal chemistry results in more rapid and optically favorable wound healing compared to the same composition crosslinked by photochemistry as well as bio-orthogonally crosslinked collagen alone. These findings underscore biochemical parameters that may be important to the success of crosslinked, in situ-forming hydrogels as an alternative to corneal transplantation, with the potential for expanded access to treatment and improved outcomes.

## Introduction

Corneal transplantation is the curative treatment for stromal scarring and opacities that result in corneal blindness, a leading cause of vision impairment that affects an estimated 12.5 million patients worldwide^1^. The first successful human corneal transplantation was conducted in 1905^2^ and has been advanced through the development of surgical microscopes, refined suture materials, the development of eye banks, the introduction of corticosteroids (to reduce graft rejection), and most recently deep anterior lamellar keratoplasty instead of full-thickness keratoplasty^3^. Nonetheless, it is still limited in several critical ways: the need for cadaveric donor tissue, which less than 2% of patients have access to; the need for highly specialized microsurgical techniques with sutures that eventually need to be removed; and the potential for graft failure^4^.

Keratoprostheses using synthetic polymers are used in refractory cases but are limited by complications related to a mismatch in material properties between rigid acrylic and native corneal tissue^5,6^. An ideal corneal substitute for human patients shares the same structure and properties as human corneal stroma. Efforts toward xenograft transplantation have been made toward this end, especially in terms of reducing the risk of zoonotic diseases and graft rejection^7–9^. A number of engineered biomaterial approaches have been reported, including both pre-formed buttons and in situ-forming constructs, with a variety of promising results^10–13^.

We have reported on the development of in situ-forming corneal stroma substitutes that have the potential to overcome the limitations faced by modern corneal transplantation^14–16^. Hydrogel pre-cursors are added into a surgically prepared corneal stromal defect as a liquid, which then forms a solid matrix within the wound and adheres to the underlying stroma without the need for sutures. While we have reported on this approach with a series of different crosslinking chemistries, we hypothesized that bio-orthogonal crosslinking chemistries with high specificity could facilitate transparent wound healing of the cornea compared to chemistries that require a catalyst, an energy source such as light, and the production of potentially harmful or inflammatory free radicals or side products^17^.

**Fig. 1:**
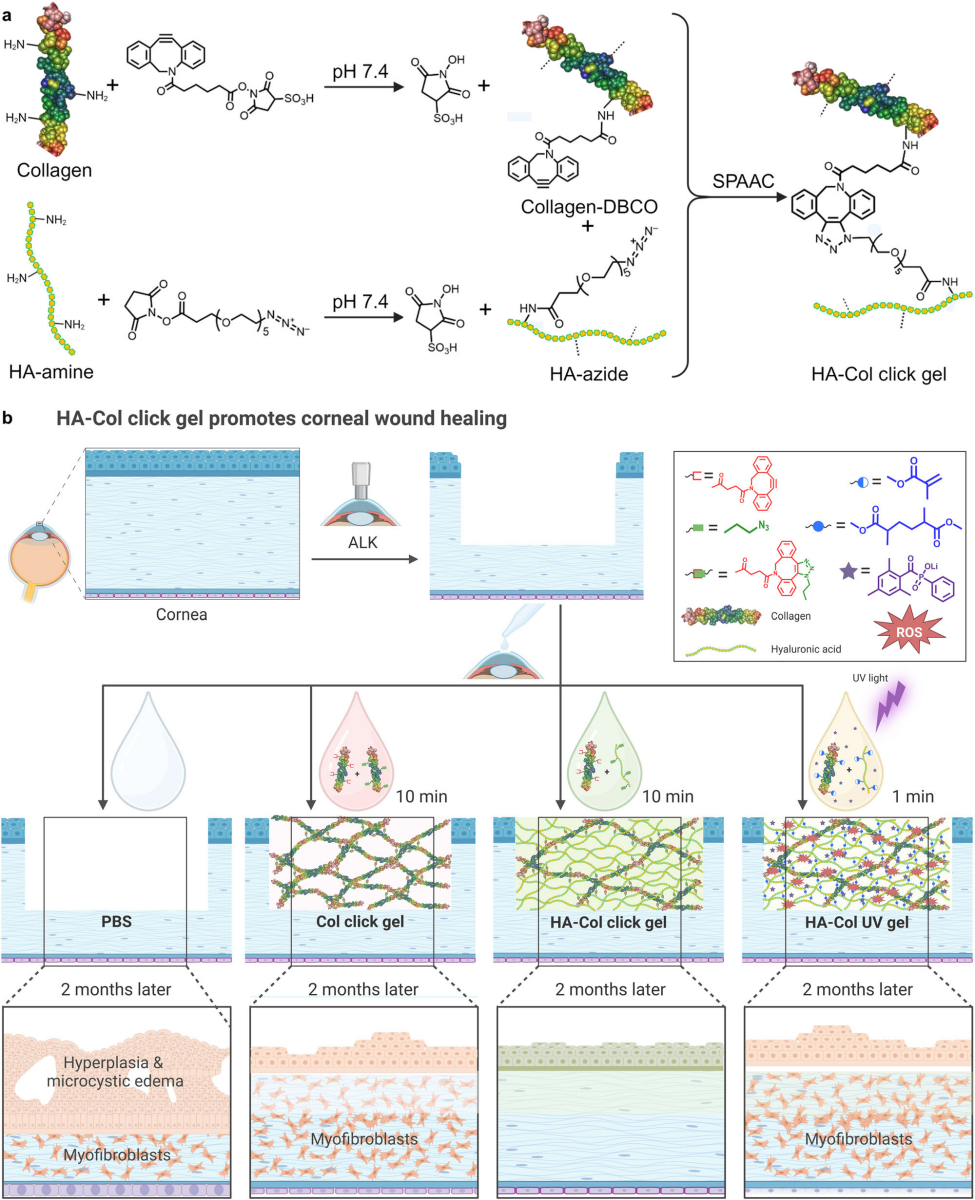
Overview of synthesis scheme for bio-orthogonally crosslinked HA-collagen (HA-Col click) gels, in vivo study design, and summary of results 2 months after treatment. **a** Collagen-DBCO and HA-azide conjugates were first synthesized and then later reacted with each other to yield the HA-Col click gels by SPAAC, a copper-free form of click chemistry. **b** The HA-Col click gels are designed to be formed in situ within keratectomy wounds. These were compared side-by-side to topical PBS alone, collagen-only gels formed in situ by SPAAC click chemistry, and HA-Col gels formed in situ by UV photopolymerization of HA-methacrylate and collagen-methacrylate conjugates. Two months after treatment, the HA-Col click gel-treated corneas exhibited more transparent and less fibrotic wound healing, including a lower degree of myofibroblastic activity within the treated stroma than the corneas treated with the HA-Col UV gel, Col click gel, and PBS. DBCO dibenzocyclooctyne, SPAAC strain promoted azide-alkyne cycloaddition, HA hyaluronic acid, Col collagen, ALK anterior lamellar keratectomy, ROS reactive oxygen species, PBS phosphate buffer solution. Created in BioRender. Lab, M. (2025) https://BioRender.com/b34n017.

To answer this question, we have directly compared the pro-regenerative and pro-inflammatory response of the wounded rabbit cornea to a composite hyaluronic acid (HA)-collagen hydrogel formed in situ with either UV-initiated free-radical crosslinking or strain-promoted azide-alkyne cycloaddition (SPAAC), a copper-free, bio-orthogonal form of click chemistry (Fig. 1a). Both forms of chemistry crosslink liquid gel precursors into solid gels within minutes. To account for the potential wound-modulating effects of HA^18–22^, we also compared these constructs to a collagen-only gel crosslinked by SPAAC.

Here, we report the first side-by-side in vivo performance of bio-orthogonally and photochemically crosslinked HA-Col gels as defect fillers after anterior lamellar keratectomy in both rat and rabbit models out to two months (Fig. 1b). The degree of corneal opacity, re-epithelialization, stroma thickness, corneal roughness, and immunohistochemical changes were closely examined post-operatively. Our results indicate that bio-orthogonal chemistry enhances epithelial and stromal regeneration while mitigating further inflammation and minimizing stromal fibrosis compared to free radical chemistry mediated by UV light exposure. They also suggest that matrix biochemistry, namely the presence of the glycosaminoglycan HA plays an important pro-regenerative and anti-inflammatory role in the context of an in situ-forming gel treatment paradigm.

## Results

### HA-Col click gels accelerated corneal re-epithelialization, decreased corneal epithelial hyperplasia, and suppressed scar formation in the corneal stroma in rats

With customized inner stopper trephines, we were able to create stromal keratectomies with consistent depth in rats^23^. The diameter of corneal wounds created in rats was 2 mm. The in situ forming gels were added to the corneal wound defects in the liquid phase. To accommodate the gel solution in the wound defect, we removed approximately 60% of the anterior cornea. Animals were randomly distributed to three treatment groups, including phosphate buffered saline (PBS), HA-Col click gel, and Col click gel. The average corneal wound depths for these groups were 66%, 62%, and 65% without significant differences between groups (Supplementary Fig. 1).

**Fig. 2:**
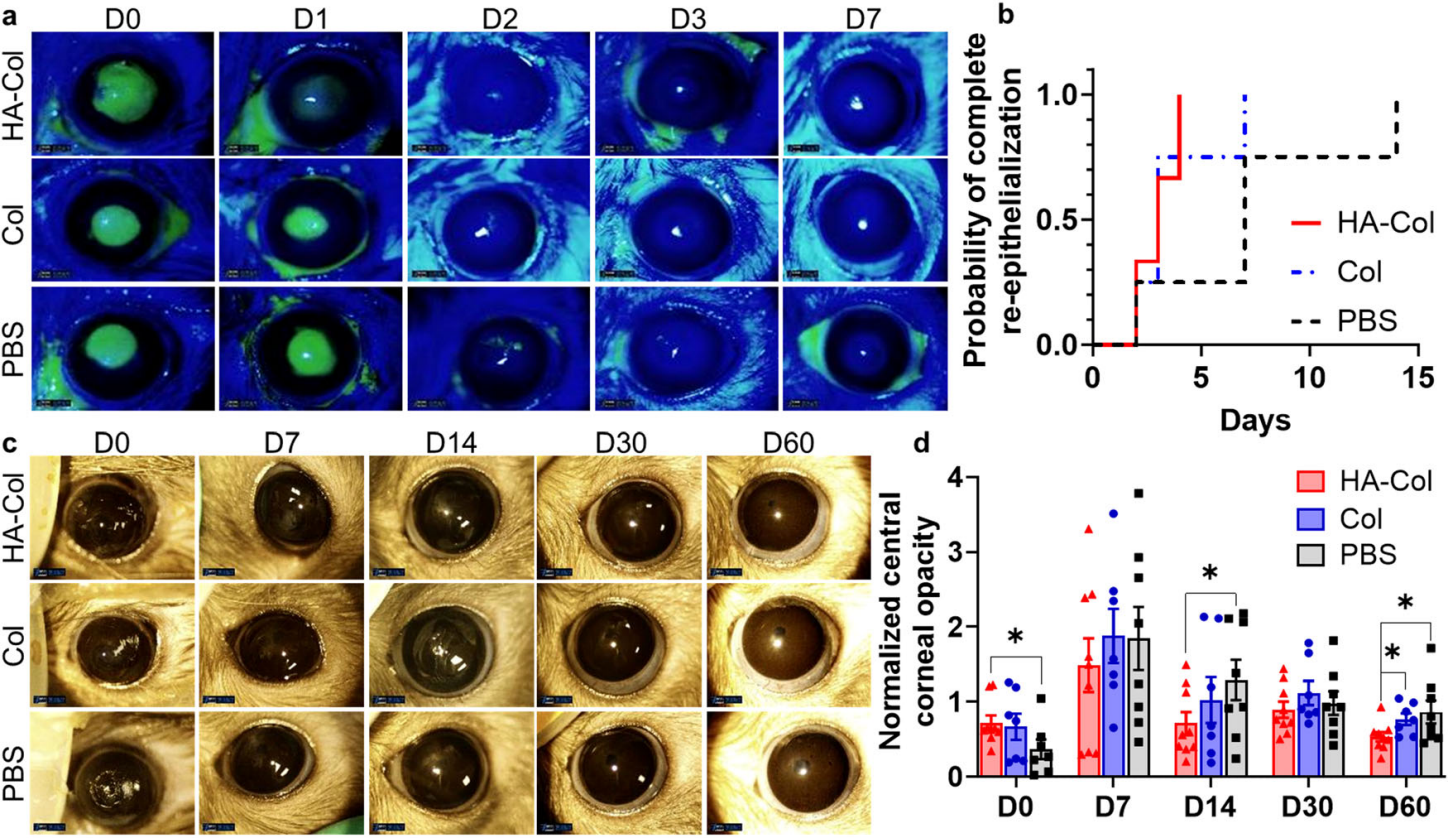
HA-Col click gels increased corneal re-epithelialization rate and decreased corneal opacity in rats. **a** Representative fluorescein staining photos of rat corneas from different treatment groups. **b** HA-Col click gel (HA-Col) treated corneas showed a higher probability of complete re-epithelialization over time compared to other groups although there were no significant differences among the treatments. *n* = 3 (PBS and HA-Col groups) or 4 (Col group). **c** Representative photos of rat corneas from different treatment groups at different time points. **d** Quantification of normalized central corneal opacity (gray value normalized to the peripheral cornea) showed the HA-Col treatment significantly decreased the corneal opacity compared to the Col click gel and PBS treatments on day 60. Data present means ± SEM. *n* = 8 (PBS group), 9 (HA-Col group), or 7 (Col group). The Heteroscedastic one-tail Student t-test was used to calculate the p values, *p < 0.05. HA is hyaluronic acid. Col is collagen. PBS is phosphate buffer solution. Graphs were created in GraphPad Prism 10.

Fluorescein staining and analysis of slit lamp images were used to determine the re-epithelialization rate of the wounded and treated corneas. All rat corneas re-epithelialized almost completely within 3 days after the treatment (Fig. 2a, b). HA-Col click gel-treated corneas showed a faster re-epithelialization compared to the other two treatments, with approximately 30% and 100% healed corneal epithelium on day 1 and day 2. Col click gel also facilitated the re-epithelialization with 22%, 96%, and 100% healed epithelium on the first three days. The PBS-treated group showed only 7%, 95%, and 99% re-epithelialization within the first three days. However, there was no significant difference in re-epithelialization among these treatments.

Opacity analysis was done with clinical photos of the eye and ImageJ. Immediately after the treatments, the PBS-treated corneas were the most transparent (Supplementary Fig. 2). Corneal opacity peaked on day 7 for all three groups. The HA-Col click gel-treated corneas became significantly more transparent than the PBS-treated ones on day 14 (Fig. 2c, d). The HA-Col click gel-treated corneas were significantly more transparent than the PBS- and Col click gel-treated ones on day 60 (Fig. 2c, d).

**Fig. 3:**
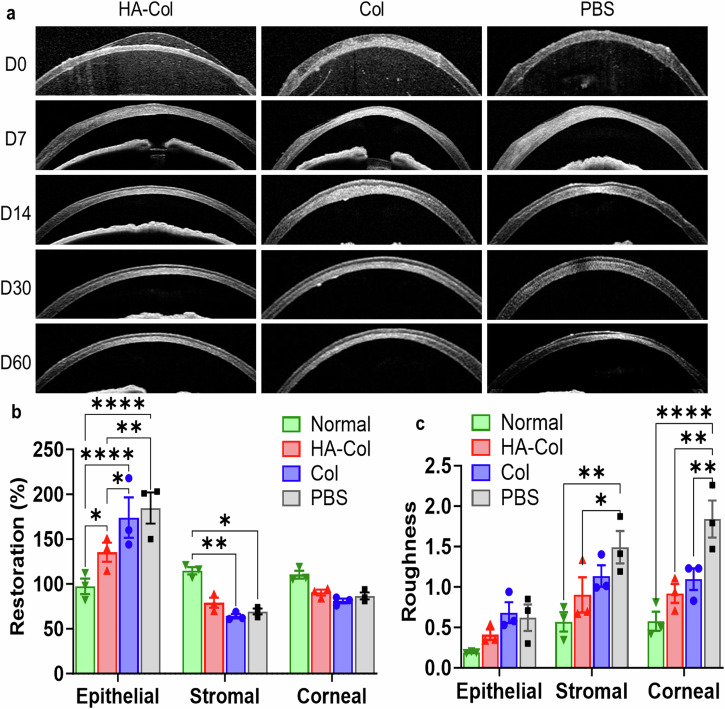
HA-Col click gels decreased corneal epithelial hyperplasia and improves stromal regeneration in rats. **a** Representative OCT images of rat corneas from different treatment groups. **b** Quantification of the normalized thickness of epithelium, stroma, and cornea on day 60. **c** Quantification of roughness of the rat’s epithelium, stroma, and cornea on day 60. Data present mean ± SEM. *n* = 3 per group. Two-way ANOVA tests were used to calculate the p values. *p < 0.05, **p < 0.01, ****p < 0.0001. HA is hyaluronic acid. Col is collagen. PBS is phosphate buffer solution. Graphs were created in GraphPad Prism 10.

The cross-sectional images of corneas were recorded using OCT imaging. We analyzed the OCT images with the maximum (central) anterior lamellar keratectomized area as shown in Fig. 3a. The epithelial/stromal/corneal thickness was evaluated by comparing the average thickness of injured epithelium/stroma/cornea and that of the adjacent intact area. After two months of treatment, the HA-Col click gel-treated corneas showed a 139%, 69%, and 82% normalized thickness in epithelial, stromal, and corneal thickness, respectively. For comparison, epithelial, stromal, and corneal thickness were 179%, 56%, and 73% in Col click gel-treated group and 190%, 60%, and 79% in PBS-treated group (Fig. 3b).

**Fig. 4:**
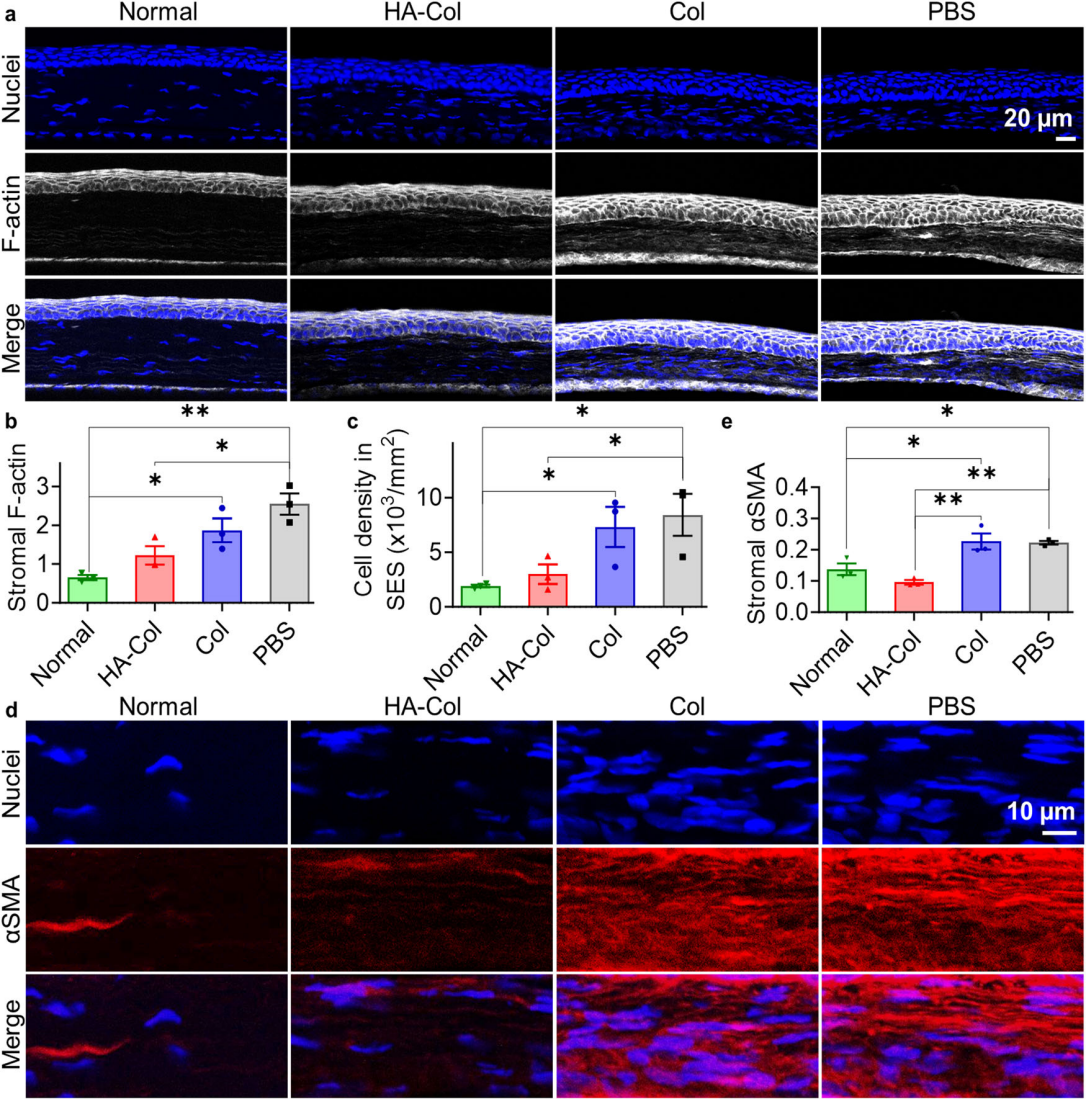
HA-Col click gels decreased scar formation associated with myofibroblastic activity in rat corneal stroma. **a** Representative F-actin staining images of rat corneas on day 60. Quantification of (**b**) stromal F-actin intensities and (**c**) cell density in the wounded corneal stroma on day 60. **d** Representative α-smooth muscle actin (αSMA) staining in the stroma of rat corneas on day 60. **e** Quantification of αSMA intensities in the stroma on day 60. All data present mean ± SEM (*n* = 3 per group). Ordinary one-way ANOVA analysis was used to calculate the *p* values. *p < 0.05, **p < 0.01. HA is hyaluronic acid. Col is collagen. PBS is phosphate buffer solution. Graphs were created in GraphPad Prism 10.

The roughness of epithelium, stroma, and cornea were quantified using the standard deviations of the epithelial, stromal, and corneal thickness from 12 evenly distributed regions of interest in the injured area. The corneal roughness of HA-Col gel-, Col gel-, and PBS-treated corneas were 1.6-, 1.9-, and 3.2-fold higher than that of normal corneas, respectively (Fig. 3c). The epithelial roughness of HA-Col gel-, Col gel-, and PBS-treated corneas were 2.1-, 3.5-, and 3.2-folds higher than that of normal epithelium, respectively. The stromal roughness of HA-Col gel-, Col gel-, and PBS-treated rats’ corneas were 1.6-, 2.0-, and 2.6-fold higher than that of normal epithelium, respectively. The representative cryosections of the corneas in Fig. 4a are highly aligned with the above quantifications.

Immunohistochemical analysis revealed that only a trace of either gel remained in the corneal stroma after 2 months (Fig. S3). The gel-treated corneas showed positive β-tubulin (a neuronal marker) staining in the corneal stroma, while the PBS-treated corneas showed negligible β-tubulin staining. The β-tubulin signal was stronger in the HA-Col click gel-treated than the Col click gel-treated corneas (Supplementary Fig. 3). Expression of stromal F-actin and α-smooth muscle actin (αSMA) was found to be high in the Col click gel- and PBS-treated corneas, while it is similar to the normal corneas in the HA-Col click gel-treated ones (Fig. 4). HA-Col click gel-, Col click gel-, and PBS-treated corneas showed an approximate 2-, 3-, and 4-fold stromal F-actin of the normal corneas (Fig. 4b). The difference between the HA-Col click gel-treated and normal corneas are insignificant, while the Col click gel- and PBS-treated groups showed significant differences in the expression of stromal F-actin when compared to the normal group. The HA-Col click gel significantly decreased the stromal F-actin expression compared to the PBS treatment.

The cell density in the corneal stroma also showed a similar trend: HA-Col click gel-, Col click gel-, and PBS-treated corneas showed an approximate 1.5-, 4-, and 4.5-fold higher stromal cell density compared to normal corneas (Fig. 4c). HA-Col click gels significantly decreased the stromal cell density when compared to the PBS treatment and showed no significant difference when compared to the normal group. HA-Col click gels also decreased the expression of stromal αSMA of wounded cornea. The signal intensity of αSMA in the HA-Col click gel-treated corneal stroma was significantly lower than in the Col click gel- and PBS-treated samples and was similar to the normal corneas (Fig. 4d, e).

**Fig. 5:**
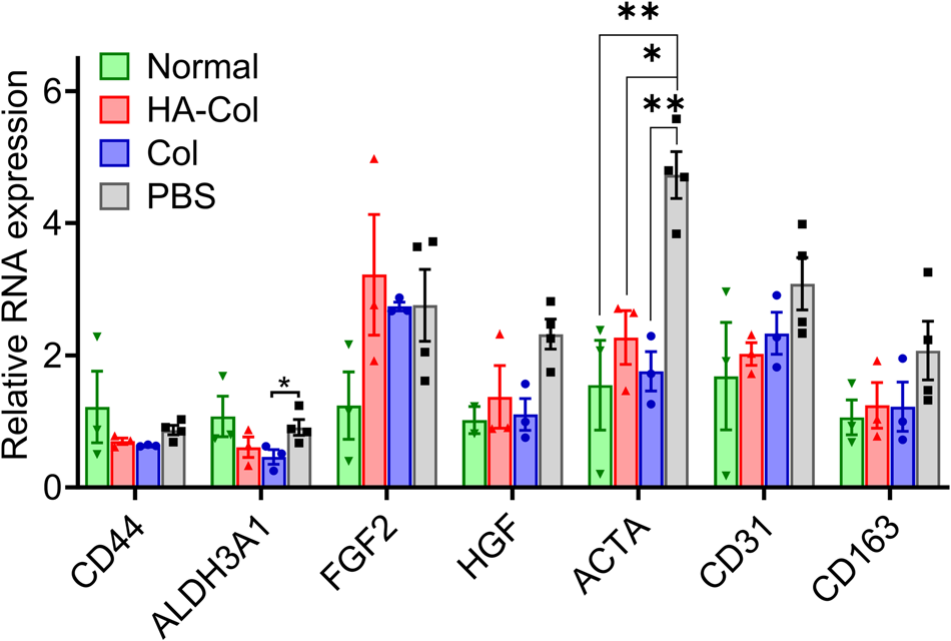
Relative RNA expression in rat corneas. PCR data showed that both gels decreased the expression of ACTA to a normal level, which was significantly lower than that of the PBS treatment. There were no significant differences among all groups in terms of the expression of CD44, ALDH3A1, FGF2, HGF, CD31, and CD163. All data present mean ± SEM (*n* = 3–4 per gene per group). Ordinary one-way ANOVA analysis was used to calculate the *p* values. *p < 0.05, **p < 0.01. HA is hyaluronic acid. Col is collagen. PBS is phosphate buffer solution. Created in GraphPad Prism 10.

Gene expression analysis showed a decreased expression of αSMA (*ACTA*) in the wounded corneas by the HA-Col click gel as well, and there was no significant difference between the HA-Col click gel-treated and normal corneas (Fig. 5). HA-Col click gel treatment downregulated the relative RNA expression of *CD44* and *ALDH3A1* and upregulated the expression of *FGF2*, *HGF*, *CD31*, and *CD163* compared to normal cornea in a non-significant manner. It significantly decreased the expression of *CD31* compared to the PBS treatment, while Col click gel treatment showed an insignificant decrease in the expression of *CD31* compared to the PBS treatment.

### HA-Col click gels reduced corneal epithelial hyperplasia and hypoplasia, prevented microcystic edema, and suppressed scar formation in the corneal stroma in rabbits

Using customized inner stopper trephines^23^, we were able to create corneal wounds with a 3.5 mm diameter and a wound depth of approximately 60% in rabbits. Animals were randomly distributed to four treatment groups: PBS, HA-Col click gel, Col click gel, and HA-Col UV gel (Supplementary Fig. 4). There was no significant difference in the cut depth among groups. The in situ forming gels (5–8 µL) were added to the corneal wound defect in liquid form and solidified in the corneal defects within 10 min.

**Fig. 6:**
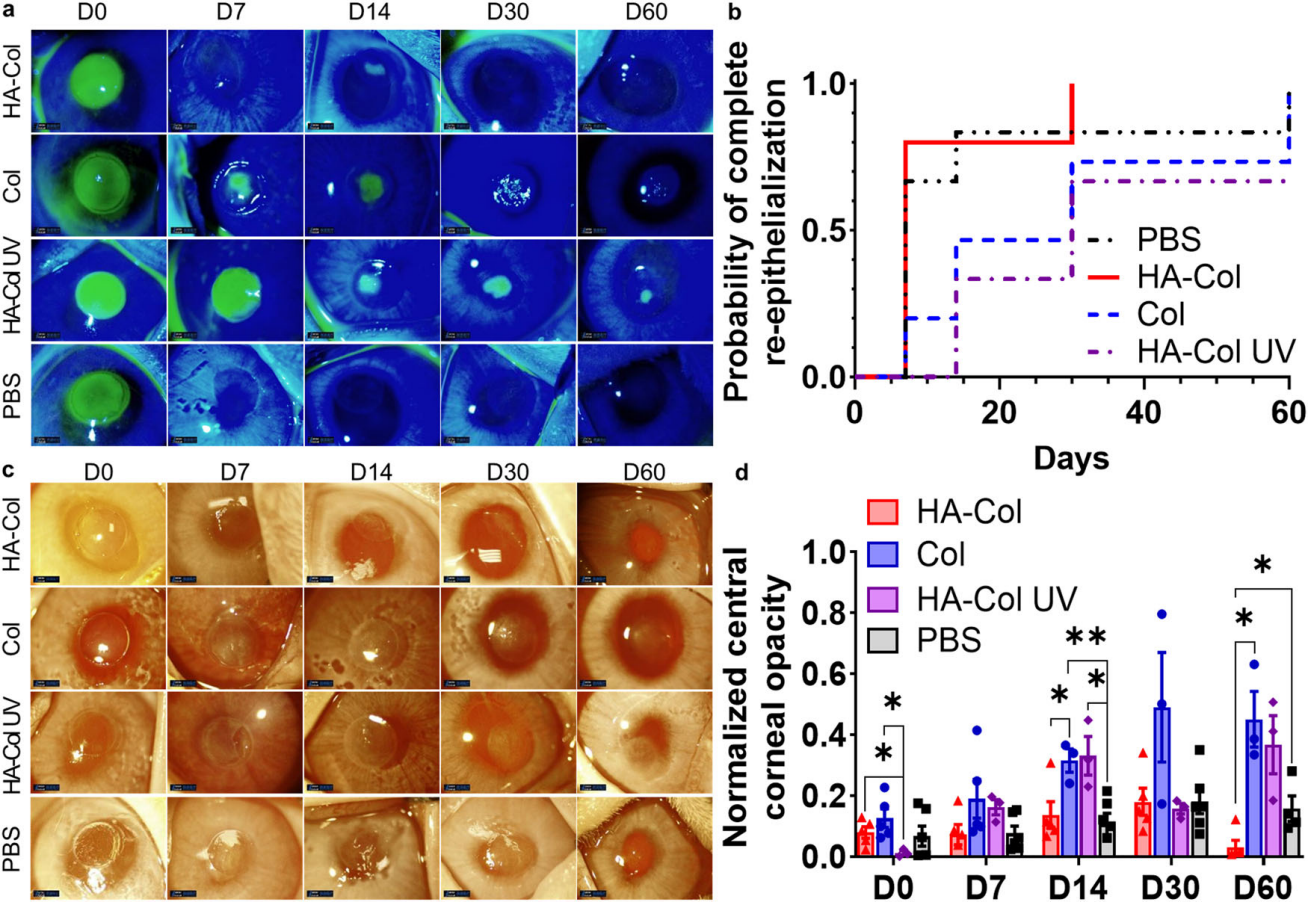
HA-Col click gels increased corneal re-epithelialization rate compared to other gels and mitigated corneal opacity in rabbits. **a** Representative fluorescein staining photos of rabbit corneas from different treatment groups. **b** HA-Col click gel treated corneas showed a higher probability of complete re-epithelialization over time compared to other groups, although there were no significant differences among the treatments. *n* = 6 (PBS), 5 (HA-Col and Col groups), or 3 (HA-Col UV group). **c** Representative photos of rabbit corneas from different treatment groups. **d** Quantification of normalized central corneal opacity (gray value normalized to the peripheral cornea) in rabbit corneas with all treatment groups. Data present mean ± SEM. *n* = 3 (Col and HA-Col UV groups), 4 (PBS group), or 5 (HA-Col group). A two-way ANOVA analysis was used to calculate the *p* values. *p < 0.05, **p < 0.01. HA is hyaluronic acid. Col is collagen. PBS is phosphate buffer solution. Graphs were created in GraphPad Prism 10.

All rabbit corneas re-epithelialized almost completely within 1 month except the HA-Col UV group, which showed a persistent small epithelial wound 2 months after the gel treatment (Fig. 6a). PBS treatment resulted in the fastest re-epithelialization rate, followed by the HA-Col click gel, Col click gel, and HA-Col UV gel treatments. The re-epithelialization in the HA-Col UV gel-treated group was significantly slower than the HA-Col and PBS treatments during the 2-month healing duration (Fig. 6b).

Opacity analysis showed that the HA-Col click gel -treated corneas resulted in significantly clearer corneas compared to PBS and other gel treatments at 2 months after surgery (Fig. 6c, d). The initial clarity of the corneas after HA-Col click gels were similar to those after Col and PBS treatment which were somewhat more edematous than the HA-Col UV gel treated corneas immediately after surgery. The corneal opacities of the eyes treated by HA-Col, Col and PBS increased and peaked at 1 month after gel treatment, whereas the opacity of HA-Col UV gel treated corneas peaked at the 2 months endpoint.

**Fig. 7:**
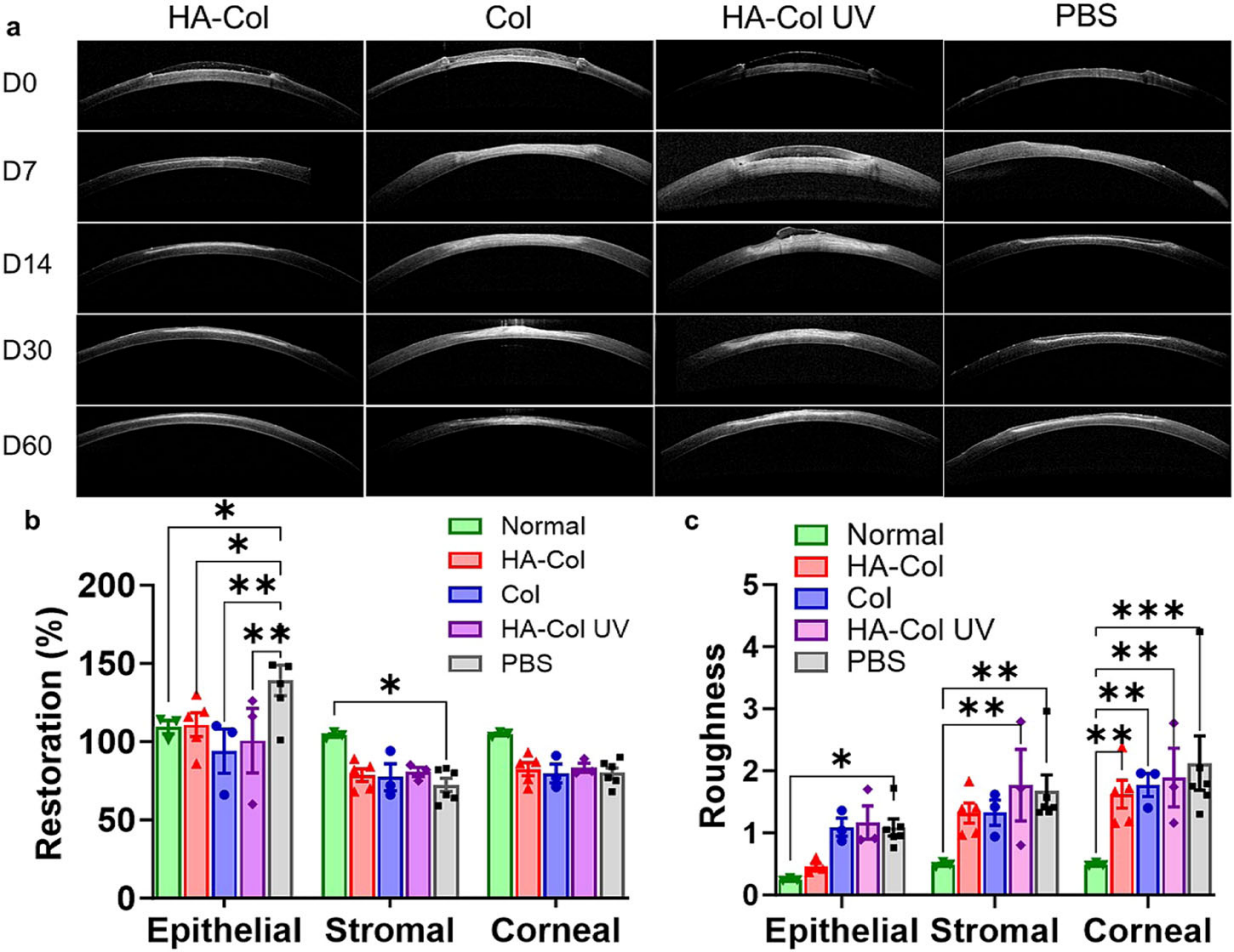
HA-Col click gels decreased the topographic irregularity of regenerated epithelia in rabbits compared to UV-crosslinked HA-Col gels. **a** Representative OCT images of rabbit corneas from different treatment groups. **b** Quantification of the normalized thickness of epithelium, stroma, and cornea thickness on Day 60. **c** Quantification of the roughness of the rabbit’s epithelium, stroma, and cornea on day 60. Data present mean ± SEM. *n* = 3 (Col and HA-Col UV groups), 4 (PBS group), or 5 (HA-Col group). Two-way ANOVA tests were used to calculate the p values. *p < 0.05, **p < 0.01. ***p < 0.001. HA is hyaluronic acid. Col is collagen. PBS is phosphate buffer solution. Graphs were created in GraphPad Prism 10.

The HA-Col click gel decreased corneal epithelial irregularity, showing less corneal epithelial hyperplasia and hypoplasia as seen in the PBS and other two gels treatment group (Fig. 7). After two months of treatment, the rabbit’s corneal epithelial thickness was restored to 101%, 86%, 92%, and 127% in HA-Col click gel-, Col click gel-, HA-Col UV gel-, and PBS-treated groups, respectively (Fig. 7a, b). The roughness of epithelia was lower in HA-Col treated corneas compared to the other injured groups (Fig. 7c). The normalized thickness of stroma and cornea was similar among all four injured groups, which were all lower than that that in normal corneas (Fig. 7). After two months of treatment, the HA-Col click gel treated rabbit corneas exhibited 79% and 76% normalized corneal and stromal thickness, which were 76% and 74% in the Col click gel-treated group, 79% and 78% in the HA-Col UV gel-treated group, and 76% and 69% in the PBS-treated group, respectively (Fig. 7a, b). The injured corneas in all treatment groups exhibited a higher roughness of both the stroma and cornea after two months of healing.

**Fig. 8:**
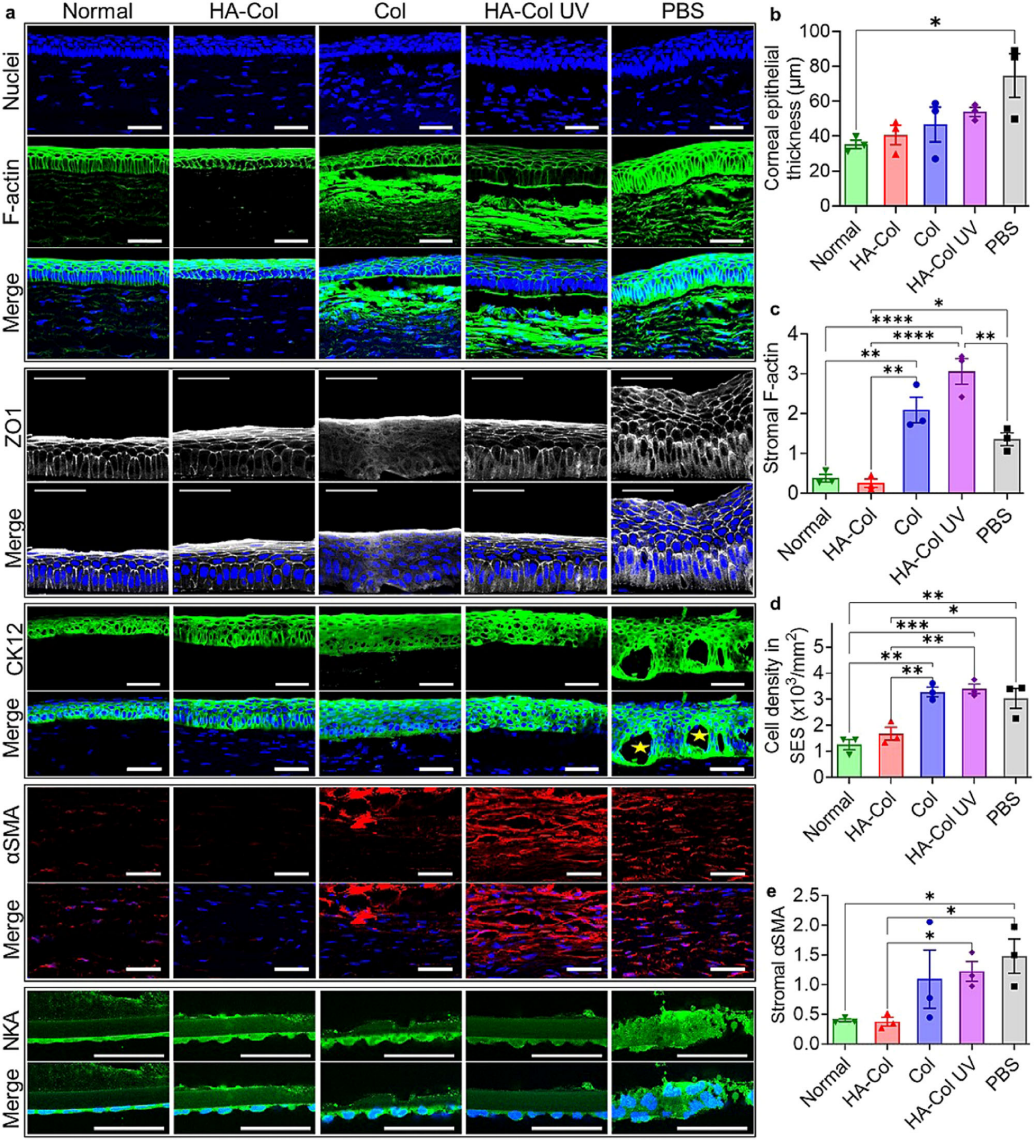
HA-Col click gels decreased scar formation associated with myofibroblastic activity in the corneal stroma and maintained the epithelial and endothelial markers in rabbit corneas. **a** Representative immunohistochemical staining images of rabbit corneas on day 60. F-actin and α-smooth muscle actin (αSMA) are biomarkers for myofibroblastic activity. Cytokeratin12 (CK12) is a biomarker for corneal epithelium. Zonula occludens (ZO)−1 is a tight junction protein in corneal epithelia. Na^+^/K^+^-ATPase (NKA) is an endothelial biomarker related to the sodium-potassium pump. Nuclei were also stained and shown in blue. Scale bars represent 50 µm in all images. Quantification of (**b**) thickness of regenerated corneal epithelia, (**c**) stromal F-actin intensities, (**d**) cell density, and (**e**) αSMA intensities in the injured corneal stroma on day 60. (E) n = 3 per group. Ordinary one-way ANOVA tests were used to calculate the *p* values. *p < 0.05, **p < 0.01, ***p < 0.001, ****p < 0.0001. HA is hyaluronic acid. Col is collagen. PBS is a phosphate buffer solution. Graphs were created in GraphPad Prism 10.

The HA-Col click gel treatment significantly decreased the expression of stromal F-actin and stromal cell density compared to the PBS, Col click, and HA-Col UV gel treatment (Fig. 8a, c, d). The expression of α-smooth muscle actin in the corneal stroma was also lower than that in the other treatments (Fig. 8a, e). The expression of stromal F-actin and α-smooth muscle actin as well as the stromal cell density in the HA-Col click gel-treated corneas were similar to normal groups. Hence, we concluded that the HA-Col click gel suppressed scar formation in the corneal stroma in rabbits.

Corneas from all injured groups showed positive staining of cytokeratin12 (CK12), a corneal epithelial biomarker, and positive staining of zonula occludens-1 (ZO-1), a tight junction protein in corneal epithelium. (Fig. 8a) Noticeably, the PBS-treated cornea had microcystic edema in the regenerated epithelia. Additionally, corneal epithelial hyperplasia was observed in the PBS-treated corneas (Fig. 8a). The corneal endothelia in all groups showed positive staining of Na^+^/K^+^-ATPase, a biomarker related to the sodium-potassium pump (Fig. 8a). The endothelial cells in the injury groups exhibited larger, hypertrophic-appearing endothelial cells. When the corneas were treated with PBS only, the monolayer of endothelial cells appeared less smooth in topography, with nuclei that were significantly larger than that of the normal, uninjured/untreated corneas. All three gels resulted in relatively less hypertrophic endothelia, while maintaining a monolayer arrangement compared to the PBS-only treatment. The HA-Col click gel maintained Descemet’s membrane thickness and morphology when compared to the Col click gel and showed a qualitatively higher expression of Na^+^/K^+^-ATPase when compared to the HA-Col UV gel.

## Discussion

Our previous studies have shown that in situ-forming hydrogels have potential as pro-regenerative stromal defect fillers in the treatment of deep corneal injuries^14–16^. Detailed synthesis methods and characterization of the HA-Col click gel have been previously reported in our published work^16^. In the context of blinding central corneal opacities, the scarred central cornea would be removed *via* anterior lamellar keratectomy and then the wound backfilled with an in situ-forming gel. With this treatment paradigm, the gel precursor must flow in and fill corneal defects, form an optically clear solid matrix within a reasonable interval, adhere and remain within the defect post-operatively, and subsequently promote epithelial wound closure and transparent stromal remodeling that results in an optically clear central cornea and smooth air-tear interface. Ideally, it would achieve all these outcomes without the need for sutures.

For such a treatment to be successful, the in situ-forming gel must promote epithelial and stromal regeneration without compromising optical clarity. The components of the gel are collagen, the primary structural protein by weight in the natural cornea, and HA, a non-sulfated extracellular matrix GAG expressed during corneal wound healing^24^. Collagen is thermosensitive and can self-assemble to form an opaque physical hydrogel. To ensure high transparency of the in situ forming hydrogels, we used chemical crosslinking and avoided physical crosslinking to form all gels. Given the reported role of endogenous HA in wound healing, we sought to answer the question of what impact HA might have on facilitating healing in our synthetic gel system, which we hypothesized would minimize inflammation and fibrosis. To test this hypothesis, we performed a direct preclinical comparison between collagen-only and collagen-HA gels. We also sought to answer the question of what impact crosslinking chemistry has on the rate and quality of corneal wound healing by comparing a widely used, conventional, free-radical mediated crosslinking approach with a photoinitiator and UV light to a highly specific, bio-orthogonal form of click chemistry that requires no energy source or catalyst and creates no by-products.

Among bio-orthogonal chemistries, strain-promoted azide-alkyne cycloaddition (SPAAC) has a number of features that make it ideal for in situ-forming chemistry directly on a corneal wound bed during surgery. Gelation kinetics are a key parameter for the translatability of in situ-forming hydrogel during eye surgery, with an ideal reaction duration between several minutes to a maximum of no more than half an hour. A reaction that is too fast would make it hard to apply and handle during an operation, while a reaction that is too slow would prolong surgery, which could be a burden to the patients, surgeons, and the healthcare system^25^. According to our previous in situ rheology studies, the HA-Col click gel starts to form before the beginning of the rheology measurements, and the storage modulus reached the half maximum in approximately 3 min and reached a final plateau in approximately 40 min^16^. In our work, we have been able to successfully mix and apply the gel to corneal wounds and complete the surgical procedure before the onset of complete gelation, as the mechanical properties of the gel in the 15–20 min range are sufficient for it to set and adhere to the cornea while continuing to react to completion underneath a soft contact lens.

In comparison, UV-crosslinking chemistry has some advantages for in situ forming hydrogel applications, particularly in terms of timing the reaction initiation, since gelation only occurs when and where there is irradiation with UV light, providing spatiotemporal control. In situ rheology tests showed that the HA-Col UV gel does not form until UV light is applied, and the storage modulus reached 70% of the maximum strength after 1 min of exposure (Supplementary Fig. 5). However, another primary determinant of an ideal in situ forming crosslinking chemistry is how the host cornea tissue responds biologically to the reaction itself, as well as any reaction sequelae. Unlike UV-crosslinking chemistry, SPAAC requires no catalyst, no energy or light source, and forms no byproducts nor any reactive radicals (Fig. 1b). Our experimental design allowed for direct comparison of the non-specific effects caused by these aspects of the UV crosslinking reaction while keeping matrix composition the same between the two gel formulations. Previously, we showed that the bio-orthogonal SPAAC crosslinked collagen click (Col click) and hyaluronan-collagen click (HA-Col click) gels promoted corneal wound healing using in vitro and ex vivo models^15,16^ but have not directly compared these to a gel of similar composition but substantially different crosslinking chemistry.

To answer the question of how matrix biochemistry (in particular, the presence or absence of HA) impacts healing, we compared a collagen-only gel (Col) to an HA-Col gel formed through identical crosslinking chemistry (using SPAAC for both). We compared the wound healing effects of these two materials first in rats, then proceeded to compare these bio-orthogonally crosslinked constructs to an HA-Col gel crosslinked by UV-activated free radical polymerization. The HA-Col and Col click gels share the same bio-orthogonal chemistry but are different in polymer content: HA-Col click gel contained 2.5% HA & 0.15% collagen, while Col click gel contained 0.3% collagen. The two HA-Col gels shared the exact same polymer content but are different in chemistry: the HA-Col click gel was crosslinked *via* SPAAC click chemistry between azide and DBCO groups, while the HA-Col UV gel was crosslinked *via* UV light-activated reaction between pendant methacrylate groups on the collagen and HA macromers.

Our results suggest that UV-activated crosslinking chemistry with free radical polymerization of methacrylate groups creates a relatively unfavorable healing environment for a keratectomized cornea stroma compared to the same composition crosslinked by bio-orthogonal chemistry. We surmise that this is the result of the additive effects of UV light, free radical production, the presence of a photoinitiator, and the generation of side products, to which the resident keratocytes must contend with and react to. We carefully controlled corneal defect diameters and depths among the treatment groups and evaluated the results real-time over 2 months using cross-sectional OCT images. There were no significant differences in the corneal defect depth among groups, making the clinical and immunohistopathological outcomes solely dependent on the treatments.

Re-epithelialization, corneal opacity, corneal thickness, corneal roughness, and biochemical changes of the regenerated corneas were examined. Corneal epithelium is able to regenerate when the limbal stem cells are functioning normally, and corneal epithelial defects could be repaired due to the migration of surrounding epithelial cells^26^. Faster corneal epithelial healing is generally desired to prevent further damage to the stroma due to, for instance, microbial infection. The HA-Col click gel accelerated the re-epithelialization rate compared to the Col click gel (Figs. 2b, 6b). This is consistent with previous preclinical experiments showing that HA promotes corneal epithelial wound healing. HA is known to induce effects on the migration, adhesion, and proliferation of corneal epithelial cells^19^. Of note, the HA-Col UV gel did not increase the re-epithelialization rate, which may be due to the production of reactive oxygen species (ROS). Excess ROS has been reported to impair corneal epithelial healing^27^ as well as to oxidize HA and therefore alter the physiological activity of HA^28^.

The enhanced re-epithelialization rate of HA-Col click gel is also supported by F-actin staining (Fig. 8a, b). Like the normal cornea, the HA-Col click gel-treated cornea exhibited higher F-actin expression in the superficial cells and polyhedral wing cells than the basal cuboidal cells. In comparison, for the PBS-treated samples, the F-actin expression was similar throughout the corneal epithelial cell layers, while in the Col click gel and HA-Col UV gel groups, the F-actin filaments were concentrated in the basal region of the cuboidal cells. These basally located actin filaments may play a role in the movement of the cells during wound healing, indicating the presence of migratory corneal epithelial cells^29,30^.

Although the rate of epithelialization was similar between the HA-Col click gel-treated corneas and the no treatment control, the HA-Col click gel significantly improved the quality and morphology of the regenerated corneal epithelia, resulting in minimal corneal epithelial hyperplasia or epithelial roughness/irregularity as well as the absence of microcystic edema (Figs. 2, 4, 7, 8). Within the underlying stroma, the HA-Col click gel treatment was found to result in decreased myofibroblast formation in the corneal stroma compared to the gel-free treatment and treatments with the HA-Col UV gel and Col-only click gel (Figs. 4, 5, 8). Consistent with this, the HA-Col click gel exhibited the lowest corneal opacity among the groups after 2 months treatment. This is significant because the formation of stromal corneal opacity despite epithelial closure after treatment would be a great impediment to patients’ vision. Minimal to no opacity is the desired outcome, as it would facilitate functional vision for patients. Corneal opacities are caused by fibroblast differentiation into myofibroblasts, which produces disorganized extracellular matrix that can scatter light. While endogenous HA synthesis mediates myofibroblast formation, exogenous HA may counter this differentiation^31^. While the potential mechanism requires future study, HA is known to interact with cells through CD44 cell-surface receptors and has been suggested to regulate the deposition of matrix proteins including fibronectin, which may promote wound healing without fibrosis^31–33^.

In addition, the HA-Col gel treated corneas resulted in a relatively healthy endothelium, while the other treatments resulted in either disrupted Descemet’s membrane, decreased endothelial cell expression of Na/K ATPase activity, or endothelial hyperplasia. Of note, all the treated and untreated corneas resulted in thinner stroma compared to native, untreated, and uninjured corneas. We postulate that the applied gels rapidly dehydrate because of endothelial cell layer pump activity upon surface epithelialization. This would explain the significant difference in thickness between the HA-Col click gel and the HA-Col UV gel at day 7 after treatment, with the latter corneas exhibiting significant stromal edema as well as gel thickness on OCT analysis (Day 7) in the context of delayed epithelialization (Fig. 6).

While the relative contributions of prolonged epithelial closure time, corneal edema, and stromal fibroblast activation are not known, the results suggest that bio-orthogonal chemistry helps to minimize each of these effects and results in a faster return to a functional, multilayered epithelium and a stromal remodeling process that is quiescent and less fibrotic, yielding treated corneas with a transparent, central optical pathway needed for vision. As the biopolymer constituents (HA and Col) of all the tested gel formulations are degraded by native enzymes, all tested gel formulations are expected to be biodegradable. Our previous study demonstrated that the HA-Col click gel is biodegradable, with 0%, 30%, 90%, and 100% of the HA-Col click gel degraded in PBS, collagenase (10 U/mL), hyaluronidase (10 U/mL), and a mixture of collagenase and hyaluronidase (both at 10 U/mL), respectively, after 24 h^16^. In vivo study showed that after 2 months of treatment with the HA-Col click gel, the corneas had only a trace of gel in the neo-stroma area, indicating re-modeling and turnover of the gel matrix within the cornea (Supplementary Fig. 3).

Further work is merited to ascertain and optimize the therapeutic potential and longer-term effects of composite HA-collagen gels formed in situ using bio-orthogonal crosslinking modalities for reconstruction and regeneration of the cornea in the treatment of deep stromal defects.

## Methods

### Study design

#### Synthesis of hydrogels

The click gels were synthesized by following our previous methods^15,16^. The HA-Col UV gel was synthesized by mixing methacrylated HA (HA-MA) and methacrylated collagen (Col-MA). HA-MA (HAMA0201, 50% substitute degree, Blafar Ltd) was reconstituted with sterile PBS to 50 mg/mL. Col-MA (5198-100MG, Advanced Biomatrix) was reconstituted to 3 mg/mL with 3 mL 20 mM acetic acid, 1.6 mL neutralization solution, and 0.4 mL 1.25% LAP solution. The 50 mg/mL HA-MA and 3 mg Col-MA containing LAP were mixed and irradiated with UltraFire 502 UV LED Flashlight (395–405 nm) for 1 min. The light intensity was controlled at 2 mW/cm^2^, which is below the known damage thresholds of UVA for the corneal endothelium, lens, and retina^34^.

#### Use of Animals

All animal experiments were designed to conform with the ARVO statement for the Use of Animals in Ophthalmic and Vision Research and were reviewed and approved by the Stanford University Institutional Animal Care and Use Committee. Female and male Rattus norvegicus (100–200 g) and Adult New Zealand white rabbits (3.5–5.5 Kg) were used in this study. For all studies, animals were housed under standard housing conditions and were acclimatized to the animal facility for 1 week before the start of the experiment. Rats were sedated with 1–4% isoflurane supplemented with 100% oxygen at a typical flow rate of 2 L/min. A cocktail of 100 mg/Kg ketamine/5 mg/Kg xylazine was injected for longer procedures. Prior to surgery, 1 drop of proparacaine hydrochloride ophthalmic solution was added to the experimental eye, and lubricant ointment was applied to the control eye. Postoperative analgesic with subcutaneous buprenorphine SR (0.5 mg/kg) was injected prior to the start of surgical procedures. Rats were euthanized by cervical dislocation under deep anesthesia. All anesthesia, analgesia, post-operation monitor, and euthanasia (IV injection of pentobarbital at 100 mg/Kg) for rabbits were performed by the veterinary service center (VSC) at Stanford University.

#### Creation of corneal wounds and gel treatment

Animals were randomly divided into experimental and control groups. Blinding is used to minimize subjective biases. In rats, partial keratectomy was performed on the right eye using a 2.0-mm customized vacuum trephines to create a deep circular cut and a spatula to remove the collagen fibril layers^35^. 2 uL of freshly made gel mixture was applied to the wound site using a pipet gun and allowed to gel in situ for 5 min. Ofloxacin ophthalmic solution was applied daily to prevent infection and to retain moisture of the eye.

In rabbits, partial keratectomy was performed on the right eye using a 3.5-mm (for rabbits) customized vacuum trephines to create a deep circular cut and a spatula to remove the collagen fibril layers. For the click gels, 5 uL of freshly made gel mixture was applied to the wound site using a pipet gun and allowed to gel in situ for 5 min. For the HA-Col UV gel, the same amount of freshly made gel mixture was applied to the wound site and irradiated with UVA light (309–405 nm) at 2 mW/cm^2^ for 1 min. The corneas were then examined with OCT and slit-lamp camera. Then, a contact lens was applied to protect the hydrogel from scratching, and a partial tarsorrhaphy was performed to prevent agitation by the animal and to help keep the contact lens and gel in place. Ofloxacin ophthalmic solution was applied daily to prevent infection and to retain moisture of the eye. The tarsorrhaphy and contact lens were removed on day 7.

#### Clinical evaluation

Treated corneas were evaluated after gel treatment and on various follow-up days for up to 2 months. Slit lamp examination and photography were used to determine corneal opacity and re-epithelialization rate. Corneal opacity was quantified by (1) comparing the gray values of the central cornea and surrounding iris area measured by ImageJ and (2) grading blindly and independently by 3 ophthalmologists following the corneal haze grading scale published previously^36^. Epithelial wound size was determined by fluorescein staining: 1–3 drops of 2.5% sodium fluorescein was placing on the ocular surface, and then washed thoroughly with plenty of Hanks’ Balanced Salt solution (BSS). Photos of cornea were then taken with irradiation of blue light. The percentile of stained green area in the entire globe area was used to determine the re-epithelialization rate. Anterior segment OCT (Spectralis® HRA + OCT w/OCT2 MultiColor model, Heidelberg) was used to evaluate the thickness and roughness of the epithelium, stroma, and cornea.

#### Immunohistochemistry

At the endpoint, the excised corneas were fixed in 4% paraformaldehyde for approximately 6 h. The fixed samples were washed with PBS thrice and then incubated overnight in the cold room with primary antibodies in 0.5% triton-x and 5% normal goat serum. Then, the slides were washed with PBS thrice and incubated with the secondary antibody solution at room temperature for 2 h. After washing, the sections were incubated with phalloidin for 30 min and then DAPI for 5 min. The sections were then mounted and imaged with a confocal microscope (Leica TCS SP5).

#### RT-qP

Corneas were used to assess gene expression 60 days post-treatment as previously described^37^. First, corneal tissues were crushed and lysed using the lyse buffer solution in the RNeasy Mini Kit (Qiagen, Hilden, Germany). RNA was purified following the manufacturer’s protocol. Next, RNA concentration was measured using NanoDrop One (Thermo Scientific), and then, a fixed amount of RNA was transcribed to cDNA using iScript Reverse Transcription Supermix for reverse transcription-quantitative polymerase chain reaction (RT-qPCR) (Bio-Rad, Hercules, CA). TaqMan Gene Expression Master Mix (Bio-Rad) was added to cDNA along with primers for *ALDH3A1*, *CD31*, *CD163*, *ACTA2*, *HGF*, and *FGF-2* (Thermo Fisher). Samples were amplified for 40 cycles using the Applied Biosystems QuantStudio 7 Flex Real-Time PCR System. Gene expression levels were compared with uninjured corneas and corneas treated with saline. *GAPDH* (Thermo Fisher) was used to normalize endogenous RNA expression within each sample. Calculation of 2^ΔCt^ was performed to give a comparative fold change of gene expression level relative to *GAPDH*.

### Statistical analysis

All data are expressed as the mean ± SEM. Statistical tests performed are specified for each figure. The ANOVA analysis was performed using GraphPad Prism 10 statistical software. Student’s t-tests were performed using Microsoft Excel 2016. *P* values smaller than 0.05 were considered significant and denoted as *p < 0.05, **p < 0.01, ***p < 0.001, and ****P < 0.0001. Outliers were excluded based on the Dixon’s Q test.

## Supplementary information

Supplemental information

## Data Availability

All data supporting the findings of this study are available from the corresponding author upon reasonable request.
