# Supplementary material for: Bio-orthogonal crosslinking and hyaluronan facilitate transparent healing after treatment of deep corneal injuries with in situ-forming hydrogels
Source: NPJ Regen Med. 2025 Feb 4;10:8. doi: 10.1038/s41536-024-00385-9 (PMC11794660; doi:10.1038/s41536-024-00385-9)
Supplement: Supplementary file 1 — Supplemental information [file 41536_2024_385_MOESM1_ESM.pdf]

Bio-Orthogonal Crosslinking and Hyaluronan Facilitate Transparent Healing after Treatment of Deep Corneal Injuries with In Situ-Forming Hydrogels

Authors: Fang Chen[†, ‡], Uiyoung Han[†, ‡], Thitima Wungcharoen[†], Youngyoon Amy Seo[†], Peter Le[†, ‡], Li Jiang[†], Nae-Won Kang[†], Euisun Song[†], Kyeongwoo Jang[†], David Mundy[†], Gabriella Maria Fernandes-Cunha[†], Sarah Heilshorn[#], David Myung[†, ‡, §, *]

Affiliations:

[†] Spencer Center for Vision Research, Byers Eye Institute, Stanford University School of Medicine, Palo Alto, CA, USA.

[‡] VA Palo Alto Health Care System; Palo Alto, CA, United States.

[§] Chemical Engineering, Stanford University, CA, United States.

* Corresponding author. Email: djmyung@stanford.edu.

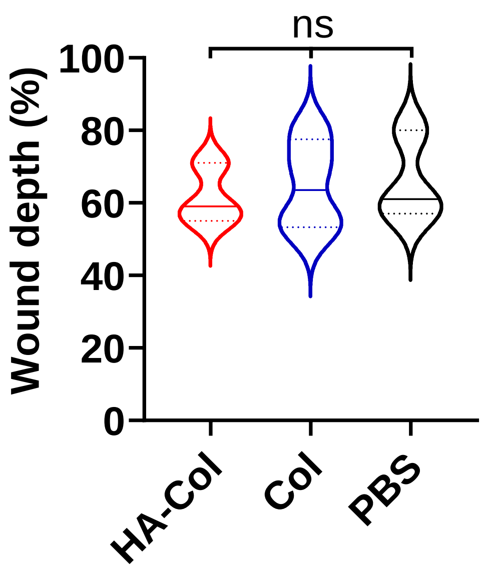

Supplementary Figure 1. Corneal wound depth in rats. The average corneal wound depths were around 64%. According to ordinary one-way ANOVA analysis, there were no significant differences in wound depths among groups. HA: hyaluronic acid, Col: collagen, PBS: phosphate buffer solution, ns: non-significant. Created in GraphPad Prism 10.

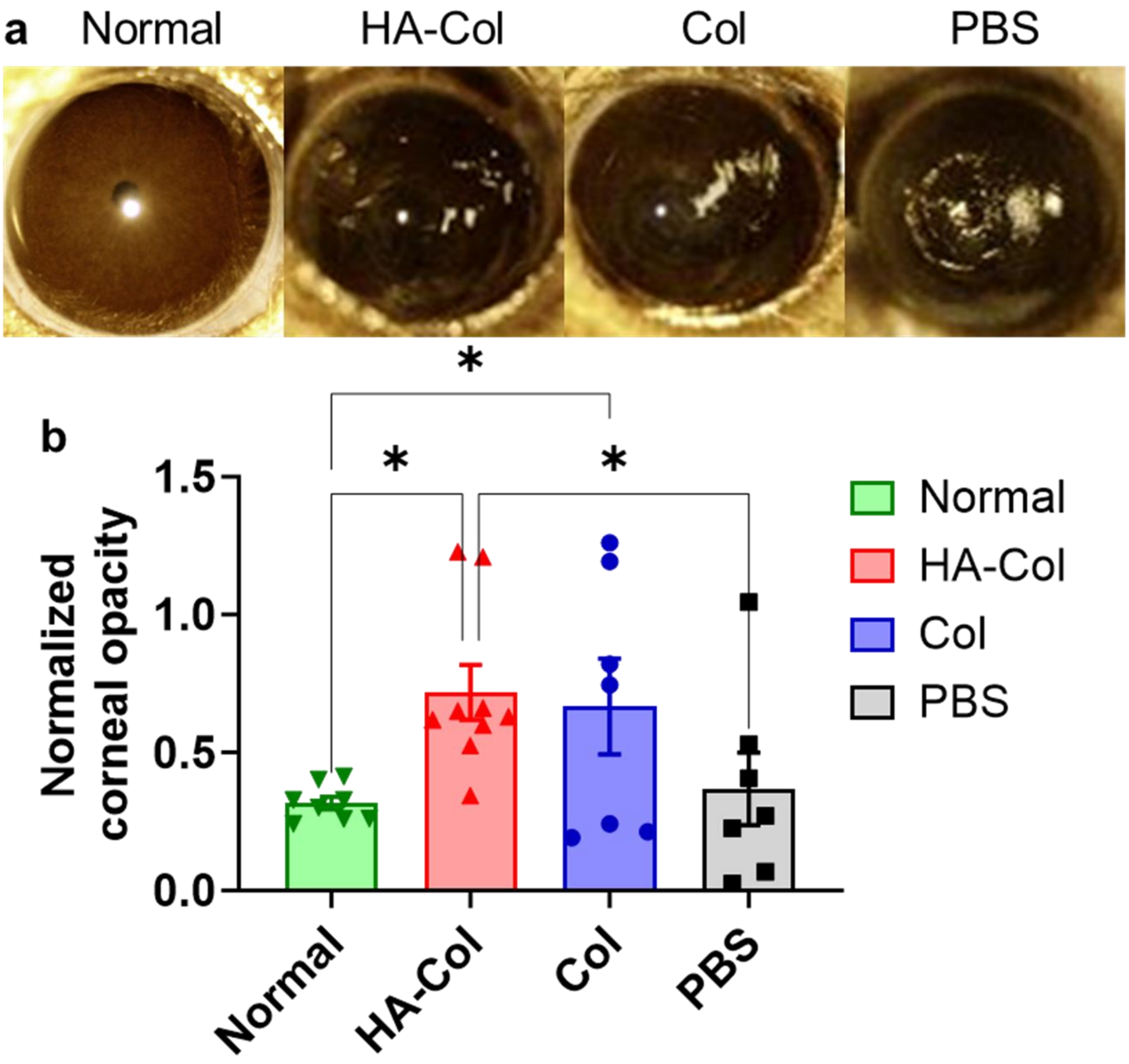

Supplementary Figure 2. HA-Col and Col click gels increased corneal opacity immediately after treatment. (A) Photo of a normal rat cornea. (B) Quantification of opacity in normal and wounded rat corneas immediately after injury and PBS or gel treatments. Data present mean ± SEM. n = 8 (normal and PBS groups), 9 (HA-Col group), or 7 (Col group). Ordinary one-way ANOVA analysis was used to calculate the p values. * $p<0.05$. HA: hyaluronic acid, Col: collagen, PBS: phosphate buffer solution, ns: non-significant. Graph was created in GraphPad Prism 10.

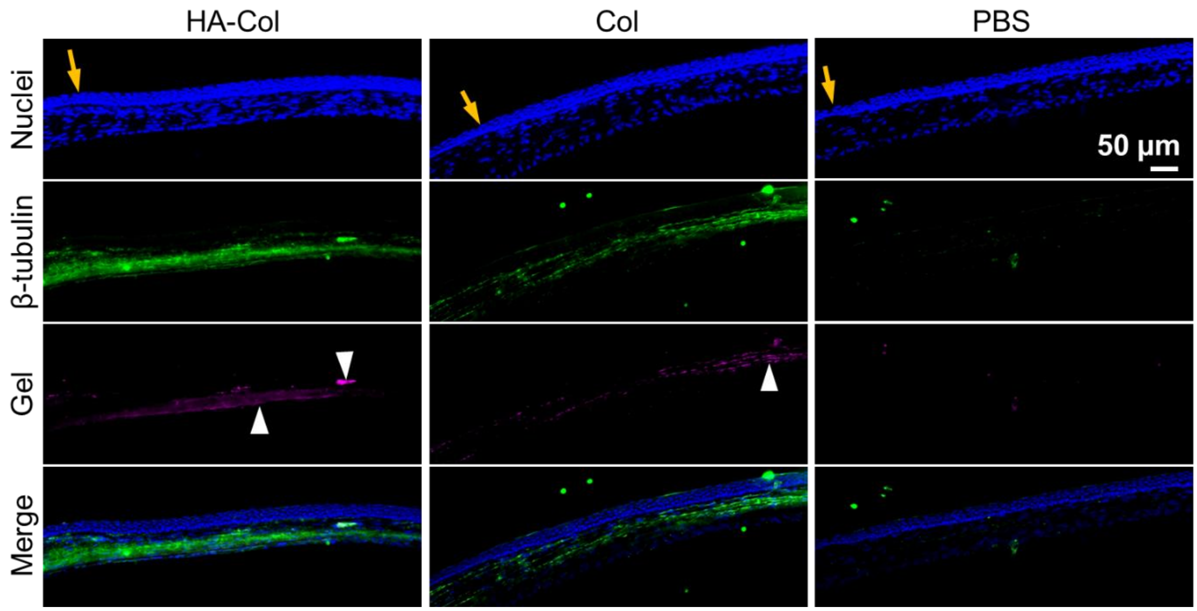

Supplementary Figure 3. Gel retention and β-tubulin staining of the rat corneas. After 2 months of treatment with gels, the corneas showed a trace of gel in the neo-stroma area. β-tubulin staining was more profound in the gels-treated groups than in the PBS-treated group. HA: hyaluronic acid, Col: collagen, PBS: phosphate buffer solution.

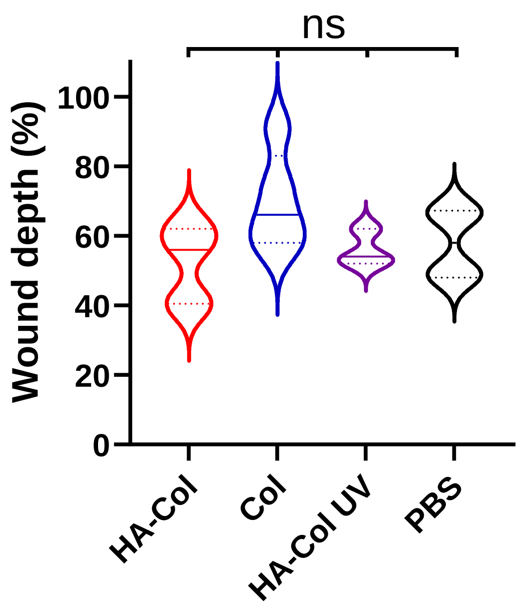

Supplementary Figure 4. Corneal wound depth in rabbits. The average corneal wound depths were around 60%. There were no significant differences in wound depth among groups according to an ordinary one-way ANOVA analysis. HA: hyaluronic acid, Col: collagen, PBS: phosphate buffer solution, ns: non-significant. Created in GraphPad Prism 10.

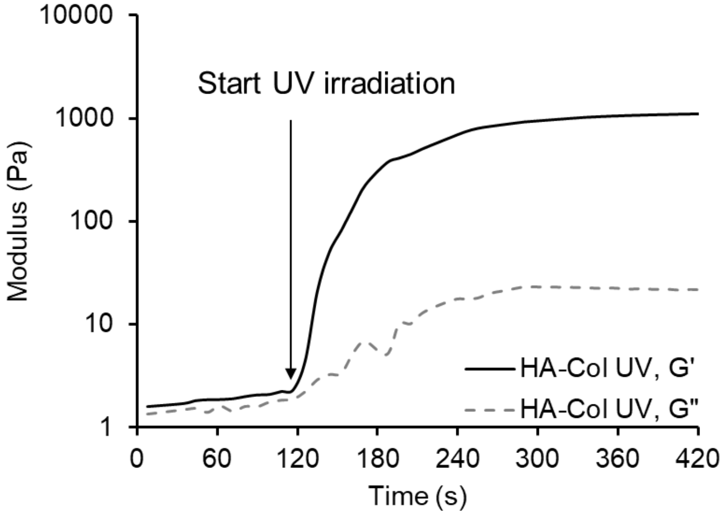

Supplementary Figure 5. In situ rheology test of HA-Col UV gel. The HA-Col UV gel formed upon UV irradiation, and the storage modulus reached 70% of the maximum strength after 1 minute UV irradiation. HA: hyaluronic acid, Col: collagen. Created in Microsoft Office 2016 Excel.
